# Evaluation of Racial Disparities in Hospice Use and End-of-Life Treatment Intensity in the REGARDS Cohort

**DOI:** 10.1001/jamanetworkopen.2020.14639

**Published:** 2020-08-24

**Authors:** Katherine A. Ornstein, David L. Roth, Jin Huang, Emily B. Levitan, J. David Rhodes, Chanee D. Fabius, Monika M. Safford, Orla C. Sheehan

**Affiliations:** 1 Department of Geriatrics and Palliative Medicine, Icahn School of Medicine at Mount Sinai, New York, New York; 2 Center on Aging and Health, Johns Hopkins School of Medicine, Baltimore, Maryland; 3 Department of Epidemiology, University of Alabama at Birmingham School of Public Health, Birmingham; 4 Department of Health Policy and Management, Johns Hopkins School of Public Health, Baltimore, Maryland; 5 Division of General Internal Medicine, Weill Cornell Medicine, New York, New York

## Abstract

**Importance:**

Although hospice use is increasing and patients in the US are increasingly dying at home, racial disparities in treatment intensity at the end of life, including hospice use, remain.

**Objective:**

To examine differences between Black and White patients in end-of-life care in a population sample with well-characterized causes of death.

**Design, Setting, and Participants:**

This study used data from the Reasons for Geographic and Racial Differences in Stroke (REGARDS) study, an ongoing population-based cohort study with enrollment between January 25, 2003, and October 3, 2007, with linkage to Medicare claims data. Multivariable logistic regression models were used to examine racial and regional differences in end-of-life outcomes and in stroke mortality among 1212 participants with fee-for-service Medicare who died between January 1, 2013, and December 31, 2015, owing to natural causes and excluding sudden death, with oversampling of Black individuals and residents of Southeastern states in the United States. Initial analyses were conducted in March 2019, and final primary analyses were conducted in February 2020.

**Main Outcomes and Measures:**

The primary outcomes of interest were hospice use of 3 or more days in the last 6 months of life derived from Medicare claims files. Other outcomes included multiple hospitalizations, emergency department visits, and use of intensive procedures in the last 6 months of life. Cause of death was adjudicated by an expert panel of clinicians using death certificates, proxy interviews, autopsy reports, and medical records.

**Results:**

The sample consisted of 1212 participants (630 men [52.0%]; 378 Black individuals [31.2%]; mean [SD] age at death, 81.0 [8.6] years) of 2542 total deaths. Black decedents were less likely than White decedents to use hospice for 3 or more days (132 of 378 [34.9%] vs 385 of 834 [46.2%]; *P* < .001). After stratification by cause of death, substantial racial differences in treatment intensity and service use were found among persons who died of cardiovascular disease but not among patients who died of cancer. In analyses adjusted for cause of death (dementia, cancer, cardiovascular disease, and other) and clinical and demographic variables, Black decedents were significantly less likely to use 3 or more days of hospice (odds ratio [OR], 0.72; 95% CI, 0.54-0.96) and were more likely to have multiple emergency department visits (OR, 1.35; 95% CI, 1.01-1.80) and hospitalizations (OR, 1.39; 95% CI, 1.02-1.89) and undergo intensive treatment (OR, 1.94; 95% CI, 1.40-2.70) in the last 6 months of life compared with White decedents.

**Conclusions and Relevance:**

Despite the increase in the use of hospice care in recent decades, racial disparities in the use of hospice remain, especially for noncancer deaths. More research is required to better understand racial disparities in access to and quality of end-of-life care.

## Key Points

**Question:**

Are there differences between Black and White patients in the use of hospice and intensity of end-of-life treatment?

**Findings:**

In this cohort study of 1212 decedents, Black individuals were significantly less likely to use hospice and more likely to have multiple emergency department visits and hospitalizations and undergo intensive treatment in the last 6 months of life compared with White individuals regardless of cause of death.

**Meaning:**

Despite the increase in the use of hospice care in recent decades, racial disparities in the use of hospice care and the intensity of end-of-life treatment remain.

## Teaser

This cohort study uses data from the Reasons for Geographic and Racial Differences in Stroke study to examine differences between Black and White patients in end-of-life care in a population sample with well-characterized causes of death.

## Introduction

Consistent with patient preferences to avoid intensive hospital-based care at the end of life,^1,2^ in 2017, most US patients died at home.^3^ Use of hospice care is on the rise in the United States—with most deaths of Medicare beneficiaries occurring while receiving hospice care, a significant increase in the last decade.^4^ The fastest-growing segment of the population receiving hospice care includes individuals with noncancer diagnoses.^5^ Despite the potential benefits of hospice enrollment for individuals and families, including enhanced quality of life for patients near the end of life^6,7,8^ and decreased Medicare costs,^9^ half of patients with terminal illnesses still do not use hospice care.^10^ Furthermore, most individuals who use hospice care are admitted very close to the end of life. Short hospice stays (≤3 days) have increased to 28.4% of all hospice stays,^10^ and 14.3% of patients with cancer who enroll in hospice do so in the last 3 days of life.^11^ In addition, high-intensity treatments at the end of life remain common despite patient preferences to avoid such care. For example, among Medicare beneficiaries, intensive care unit use in the last month of life increased from 24% in 2000 to 29% in 2015.^4^

Research has documented racial differences in hospice use and end-of-life treatment intensity, consistent with a broad range of racial disparities in health care use and health outcomes. In general, Black decedents receive more aggressive care, have higher end-of-life health care spending, and are less likely to use hospice services than White decedents.^12,13,14^ Reasons for these disparities include preferences for more aggressive care, mistrust of the health care system, lack of in-home resources, and miscommunication and misunderstanding of treatment options.^15^

Because substantial shifts in end-of-life health care delivery have occurred, it is critical to better understand whether similar racial differences persist in the setting of different diseases. We used the Reasons for Geographic and Racial Differences in Stroke (REGARDS) study linked to Medicare claims data to examine disparities in end-of-life care among individuals who have died of stroke, heart disease, cancer, dementia, or other serious illnesses. We hypothesized that high-intensity treatments at the end of life will be more common among Black than White decedents and that Black decedents will be less likely to use hospice care regardless of the cause of death.

## Methods

### Data Sources and Study Population

The data for this study are from REGARDS, an ongoing population-based cohort study of racial and regional differences in stroke mortality, with oversampling of Black individuals and residents of Southeastern states in the United States. The recruitment, enrollment, and assessment procedures of this national, population-based study are described in detail elsewhere.^16^ A total of 30 239 adults 45 years or older were enrolled between January 25, 2003, and October 3, 2007. Exclusion criteria included residence in or on a waiting list for a nursing home; cancer diagnosis requiring active treatment, such as chemotherapy or radiotherapy; and not speaking English. Every 6 months, follow-up telephone interviews are conducted with REGARDS enrollees to inquire about hospitalizations, outpatient visits, and any symptoms that might indicate possible study end points (eg, stroke and myocardial infarction). All REGARDS participants signed written informed consent statements that authorized the investigators to access their medical records to detect possible strokes, myocardial infarctions, and other medical events. This included permission to access electronic and administrative medical records, including Medicare claims. Medicare claims files have been successfully linked to REGARDS participants^17^ and examined in previous analyses of health care use.^18,19^ The REGARDS participants who died between January 1, 2013, and December 31, 2015, were included in this study, and the study was reviewed and approved by the institutional review boards of the University of Alabama at Birmingham and Johns Hopkins University. This report followed the Strengthening the Reporting of Observational Studies in Epidemiology (STROBE) reporting guideline.^20^

### Outcome Measures

Our primary outcomes of interest were hospice use and other measures of end-of-life treatment intensity within the last 6 months of life.^21^ All outcome variables were derived from Medicare fee-for-service (parts A and B) claims files. We focused on hospice use of 3 or more days as our primary measure to avoid capturing suboptimal hospice use owing to short duration.^7^ Next, building on previous work,^22,23,24^ we identified the use of 1 or more intensive life-sustaining medical procedures as determined by a review of *International Classification of Diseases, Ninth Revision* (*ICD-9*) codes or *International Statistical Classification of Diseases and Related Health Problems, Tenth Revision* (*ICD-10*) codes in each decedent’s Medicare claims. These procedures included intubation and mechanical ventilation, tracheostomy, gastrostomy tube insertion, enteral or parenteral nutrition, hemodialysis, and cardiopulmonary resuscitation. Finally, we counted the number of emergency department (ED) admissions and inpatient hospitalizations in the 6-month period before death. Our primary focus was 2 or more ED visits and 2 or more hospital stays.

### Main Exposure

Race in REGARDS is determined by self-report. Because the REGARDS study was designed to examine the factors that might explain the increased stroke mortality previously observed for Black individuals, only Black individuals and White individuals, who served as the reference group, were enrolled, and individuals who self-identified as Hispanic, Latino, Asian, or other races/ethnicities were excluded.

### Covariates

Cause of death in the REGARDS study is adjudicated by an expert panel of clinicians using methods that are consistent with American Heart Association consensus guidelines.^25,26^ Death certificates, autopsy reports, and medical records from hospitalizations in the last 6 months of life were retrieved. Participant proxies (next of kin, family members, or close friends) were also interviewed to obtain information about the events surrounding the death. Each death is adjudicated independently by 2 expert clinicians who conduct their reviews using all information available at the time of review, including the participant’s clinical baseline characteristics, death certificates, proxy interviews, the National Death Index, and medical records from recent hospital admissions. After individual review, the adjudicators agree on the underlying cause of death. Disagreements are resolved by consensus. Because of our interest in the decision to use hospice services, we excluded individuals who were determined by the adjudication panel to have experienced sudden death or death due to unnatural causes (10.8% [146 of 1358] of all deaths). For the present analyses, we grouped cause of death into the following 4 categories: cancer (all types); dementia; cardiovascular disease (CVD), including myocardial infarction, heart failure, stroke, and pulmonary embolism; and other, which included causes such as end-stage kidney disease, liver disease, infection, respiratory disease, and other medical problems not considered to be sudden deaths.

Demographic variables (sex, date of birth, marital status, annual household income, and educational level) were collected from a computer-assisted telephone interview conducted by trained interviewers at the time of enrollment into REGARDS. Location of residence was categorized based on census region and whether it was in the southern “stroke belt” region of the United States (1 of 8 states [North Carolina, South Carolina, Georgia, Tennessee, Alabama, Mississippi, Arkansas, and Louisiana] with high stroke mortality). The Charlson Comorbidity Index score was calculated using *ICD-9* and *ICD-10* diagnosis codes from the inpatient, outpatient, and physician claim (carrier) files for the 6-month period preceding death. These codes were then mapped to 17 comorbid conditions, weights were assigned, and the index was calculated.^19^ Medicaid dual-eligibility status was obtained from the Medicare beneficiary enrollment file.

### Statistical Analysis

Initial analyses were conducted in March 2019, and final primary analyses were conducted in February 2020. Based on a conceptual framework of treatment intensity for patients with serious illness^27^ and a review of the literature, we first compared key clinical and demographic characteristics and markers of end-of-life treatment intensity by race. We used the χ^2^ test, the *t* test, and the Wilcoxon rank sum test as appropriate. After stratifying by cause of death, we examined racial differences in markers of end-of-life treatment intensity. Next, we used multivariable logistic regression to examine the association of race with each of 4 outcomes: (1) hospice use of 3 or more days, (2) multiple (≥2) ED visits in the last 6 months of life, (3) multiple (≥2) hospital stays in the last 6 months of life, and (4) any use of intensive treatments in the last 6 months of life. Each model was adjusted for cause of death, sex, marital status, educational level, income, Charlson Comorbidity Index score, age at death, and Medicaid status. We also tested for interactions by race and cause of death for each outcome. SAS statistical software version 9.4 was used for all analyses (SAS Institute), and *P* < .05 was considered statistically significant. All tests were 2-tailed.

## Results

We identified 1212 participants (630 men [52.0%]; 378 Black individuals [31.2%]; mean [SD] age at death, 81.0 [8.6] years) who constituted the study sample (Table 1). A total of 610 participants (50.3%) were married at the time of death, and 200 (16.5%) had less than a high school education. Decedents who died prior to 2013 were excluded owing to the unavailability of data on hospice use. Furthermore, decedents in REGARDS with no Medicare claims or who were enrolled only in Medicare part C were excluded. Among the 1358 REGARDS participants who died during the period from 2013 to 2015 with Medicare fee for service (parts A and B coverage) in the last 6 months of life, we further excluded 83 who had adjudicated sudden cause of death (eg, cardiac sudden deaths) and 54 who died owing to an accident, injury, suicide, or homicide. Among the remaining 1221 decedents, we excluded 9 with an undetermined cause of death and included 1212 with cause-of-death adjudication in the present analyses (eFigure in the Supplement).

**Table 1.** Demographic and Clinical Characteristics of REGARDS Decedents by Race

Variable	No. (%)			*P* value
	Overall (N = 1212)	White (n = 834)	Black (n = 378)	
Age at REGARDS baseline, mean (SD), y	71.4 (8.5)	72.2 (8.2)	69.8 (9.1)	<.001
Age at death, mean (SD), y	81.0 (8.6)	81.7 (8.2)	79.5 (9.2)	<.001
Female sex	582 (48.0)	366 (43.9)	216 (57.1)	<.001
Educational level				
<High school	200 (16.5)	92 (11.0)	108 (28.6)	<.001
High school graduate	331 (27.3)	228 (27.3)	103 (27.3)	
Some college	323 (26.7)	227 (27.2)	96 (25.4)	
College graduate and above	358 (29.5)	287 (34.4)	71 (18.8)	
Income, $				
<20 000	247 (20.4)	117 (14.0)	130 (34.4)	<.001
20 000-34 000	357 (29.5)	247 (29.6)	110 (29.1)	
35 000-74 000	312 (25.7)	256 (30.7)	56 (14.8)	
≥75 000	113 (9.3)	96 (11.5)	17 (4.5)	
Refused	183 (15.1)	118 (14.2)	65 (17.2)	
Marital status				
Married	610 (50.3)	476 (57.1)	134 (35.5)	<.001
Single	44 (3.6)	20 (2.4)	24 (6.4)	
Widowed	408 (33.7)	263 (31.5)	145 (38.4)	
Divorced	134 (11.1)	73 (8.8)	61 (16.1)	
Other	16 (1.3)	2 (0.2)	14 (3.7)	
Has Medicaid	253 (20.9)	97 (11.6)	156 (41.3)	<.001
Charlson Comorbidity Index score, mean (SD)	4.4 (3.1)	4.1 (3.0)	5.1 (3.1)	<.001
Depression measured via CES-D score, mean (SD)	4.7 (4.7)	4.5 (4.6)	5.2 (5.0)	.02
Long-term care placement	289 (23.8)	176 (21.1)	113 (29.9)	<.001
Cause of death				
CVD	297 (24.5)	195 (23.4)	102 (27.0)	.16
Cancer	309 (25.5)	228 (27.3)	81 (21.4)	
Dementia	115 (9.5)	78 (9.4)	37 (9.8)	
Other illness	491 (40.5)	333 (39.9)	158 (41.8)	
Lives alone	429 (35.4)	282 (33.8)	147 (38.9)	.09
Available caregiver	971 (80.1)	680 (81.5)	291 (77.0)	.07
Stroke belt^a^	710 (58.6)	526 (63.1)	184 (48.7)	<.001
Census region				
Northeast	76 (6.3)	46 (5.5)	30 (7.9)	<.001
Midwest	188 (15.5)	105 (12.6)	83 (22.0)	
South	874 (72.1)	628 (75.3)	246 (65.1)	
West	74 (6.1)	55 (6.6)	19 (5.0)	

Abbreviations: CES-D, Center for Epidemiological Studies–Depression; CVD, cardiovascular disease; REGARDS, Reasons for Geographic and Racial Differences in Stroke.

^a^ Residence in one of the following 8 states with high stroke mortality: North Carolina, South Carolina, Georgia, Tennessee, Alabama, Mississippi, Arkansas, or Louisiana.

As shown in Table 1, Black decedents were 2 years younger than White decedents at the time of death (mean [SD] age, 79.5 [9.2] vs 81.7 [8.1] years). Consistent with previously reported demographic differences for the REGARDS sample,^16^ Black participants in this analysis were less educated than White participants (less than high school education, 108 of 378 [28.6%] vs 92 of 834 [11.0%]) and more likely to have Medicaid dual eligibility (156 of 378 [41.3%] vs 97 of 834 [11.6%]). Black decedents also had more comorbidities at the time of death than White decedents (mean [SD] Charlson Comorbidity Index sccore, 5.1 [3.1] vs 4.1 [3.0]). Cause of death was determined as dementia, CVD, or cancer for 220 of 378 Black decedents (58.2%) and 501 of 834 White decedents (60.1%). The remaining causes of death were grouped together as other illness.

Black decedents were less likely than White decedents to use hospice for 3 or more days (132 of 378 [34.9%] vs 385 of 834 [46.2%]; *P* < .001) (Table 2). Black decedents also had a higher number of repeated hospitalizations and ED visits in the last 6 months of life than White decedents; 224 of 378 Black decedents (59.3%) were seen in the ED more than once in the last 6 months of life vs 387 of 834 White decedents (46.4%). Of 378 Black decedents, 190 (50.3%) had more than 1 hospitalization in the last 6 months of life compared with 300 of 834 White decedents (36.0%). For intensive procedures, Black decedents generally were 2 or more times as likely as White decedents to have received each procedure in the last 6 months of life. For example, 80 of 378 Black decedents (21.2%) were intubated or had mechanical ventilation compared with 94 of 834 White decedents (11.3%).

**Table 2.** End-of-Life Health Care Use in the Last 6 Months of Life, Overall and by Race

Variable	No. (%)			*P* value
	Overall (N = 1212)	White (n = 834)	Black (n = 378)	
Hospice				
Any hospice use	643 (53.1)	479 (57.4)	164 (43.4)	<.001
Hospice use ≥3 d	517 (42.7)	385 (46.2)	132 (34.9)	<.001
Total No. of days in hospice, median (IQR)^a^	15.00 (5.00-59.00)	13.00 (5.00-59.00)	17.00 (5.00-58.50)	.35
Healthcare use				
ED visits	956 (78.9)	641 (76.9)	315 (83.3)	.01
≥2 ED visits	611 (50.4)	387 (46.4)	224 (59.3)	<.001
No. of ED visits, mean (SD)^a^	2.5 (1.7)	2.3 (1.6)	2.8 (1.8)	<.001
Hospital stays	894 (73.8)	593 (71.1)	301 (79.6)	.002
≥2 Hospital stays	490 (40.4)	300 (36.0)	190 (50.3)	<.001
No. of hospital stays, mean (SD)^a^	2.0 (1.2)	1.9 (1.1)	2.2 (1.3)	<.001
Intensive procedures				
Intubation or mechanical ventilation	174 (14.4)	94 (11.3)	80 (21.2)	<.001
Tracheostomy	17 (1.4)	5 (0.6)	12 (3.2)	<.001
Gastrostomy tube	51 (4.2)	22 (2.6)	29 (7.7)	<.001
Hemodialysis	70 (5.8)	27 (3.2)	43 (11.4)	<.001
Enteral or parenteral nutrition	60 (5.0)	29 (3.5)	31 (8.2)	<.001
CPR	53 (4.4)	22 (2.6)	31 (8.2)	<.001
Any intensive procedures	279 (23.0)	148 (17.8)	131 (34.7)	<.001

Abbreviations: CPR, cardiopulmonary resuscitation; ED, emergency department; IQR, interquartile range.

^a^ Among those with any ED visits or hospital stays.

After stratifying by cause of death, we found substantial racial differences in treatment intensity among those with CVD deaths (Figure). Although there were no significant differences in hospice use, Black participants who died of CVD were significantly more likely than White decedents to have more than 1 ED visit (68 of 102 [66.7%] vs 87 of 195 [44.6%]) and multiple hospital stays (53 of 102 [52.0%] vs 61 of 195 [31.3%]) and to have undergone intensive procedures (38 of 102 [37.3%] vs 34 of 195 [17.4%]). Similarly, among other deaths not attributed to cancer, dementia, or CVD, Black decedents were less likely to use hospice care (36 of 158 [22.8%] vs 120 of 333 [36.0%]) and significantly more likely than White decedents to have multiple ED visits (101 of 158 [63.9%] vs 154 of 333 [46.2%]) and multiple hospital stays (85 of 158 [53.8%] vs 129 of 333 [38.7%]) and to have undergone intensive procedures (70 of 158 [44.3%] vs 78 of 333 [23.4%]). Among cancer deaths, we did not observe statistically significant racial differences in treatment intensity among any indicators. Among participants whose cause of death was dementia, there was no significant difference in hospice use, ED visits, or hospitalizations by race, although Black decedents were significantly more likely to undergo intensive procedure use (6 of 37 [16.2%] vs 2 of 78 [2.6%]).

**Figure.**
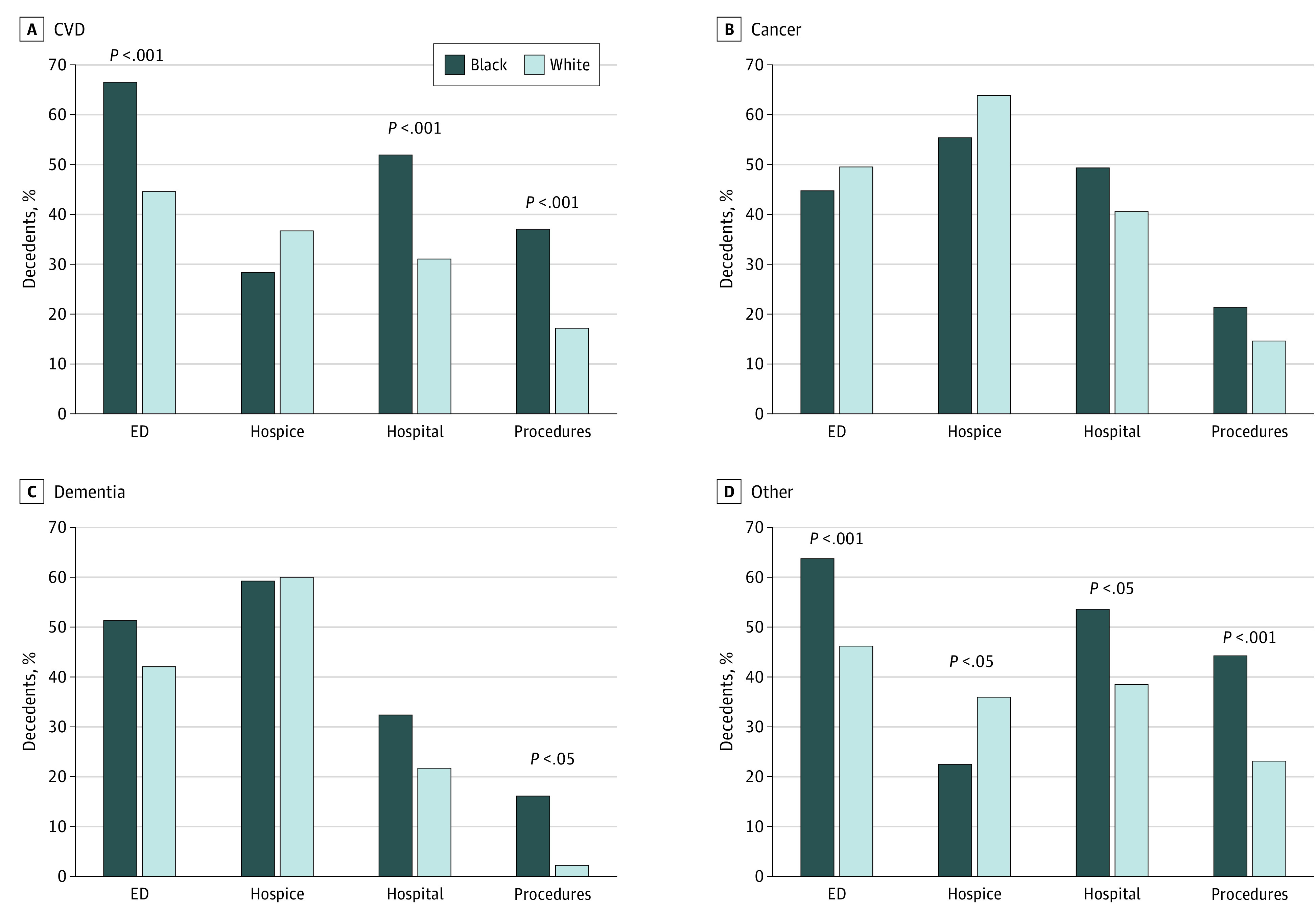
End-of-Life Health Care Use in the Last 6 Months of Life by Race and Cause of Death CVD indicates cardiovascular disease; ED, emergency department.

Our covariate-adjusted models are summarized in Table 3. Of primary interest is the association of race with outcomes after adjusting for covariates. Significant racial differences were found for all 4 outcomes (hospice use ≥3 days, multiple ED visits, multiple hospital stays, and intensive procedure use in the last 6 months of life). Black decedents were less likely than White decedents to use hospice (adjusted odds ratio [aOR], 0.72; 95% CI, 0.54-0.96) and more likely to use all other forms of health care in the last 6 months of life. In particular, Black decedents were more likely to have multiple ED visits (aOR, 1.35; 95% CI, 1.01-1.80) and hospitalizations (aOR, 1.39; 95% CI, 1.02-1.89) and had nearly twice the odds of undergoing intensive procedures in the last 6 months of life (aOR, 1.94; 95% CI, 1.40-2.70).

**Table 3.** Association of Race With Hospice Use, Multiple ED Visits, Multiple Hospitalizations, and Intensive Procedure Use in the Last 6 Months of Life

Variable	Odds ratio (95% CI)^a^							
	Hospice use ≥3 d		≥2 ED visits		≥2 Hospital visits		Any intensive procedures	
	Model 1	Model 2	Model 1	Model 2	Model 1	Model 2	Model 1	Model 2
Race, Black vs White	0.63 (0.49-0.81)	0.72 (0.54-0.96)	1.68 (1.31-2.15)	1.35 (1.01-1.80)	1.80 (1.41-2.30)	1.39 (1.02-1.89)	2.46 (1.87-3.24)	1.94 (1.40-2.70)
Cause of death^b^								
CVD	NA	0.25 (0.18-0.36)	NA	2.00 (1.38-2.88)	NA	1.78 (1.20-2.64)	NA	2.92 (1.86-4.60)
Dementia	NA	0.62 (0.38-1.01)	NA	2.29 (1.39-3.79)	NA	1.74 (0.98-3.08)	NA	1.12 (0.48-2.59)
Other illness	NA	0.23 (0.17-0.32)	NA	2.00 (1.44-2.78)	NA	2.33 (1.63-3.35)	NA	3.97 (2.64-5.98)
Female sex	NA	1.25 (0.95-1.65)	NA	1.19 (0.90-1.58)	NA	1.61 (1.18-2.18)	NA	1.16 (0.83-1.62)
Age at death	NA	1.03 (1.02-1.05)	NA	1.01 (1.00-1.03)	NA	1.00 (0.99-1.02)	NA	0.95 (0.93-0.97)
Educational level,≥college vs ≤ college	NA	0.96 (0.73-1.27)	NA	0.80 (0.60-1.05)	NA	0.92 (0.68,1.26)	NA	0.91 (0.64-1.29)
Marital status: married vs other	NA	1.21 (0.91-1.61)	NA	1.06 (0.79-1.41)	NA	1.17 (0.85-1.60)	NA	1.16 (0.82-1.64)
Income, $								
≥75 000 vs <75 000	NA	1.06 (0.69-1.65)	NA	1.24 (0.79-1.94)	NA	0.90 (0.55-1.46)	NA	0.83 (0.48-1.43)
Refused vs <75 000	NA	0.75 (0.53-1.07)	NA	0.80 (0.56-1.13)	NA	0.73 (0.50-1.08)	NA	0.92 (0.60-1.40)
Dual eligible for Medicare and Medicaid	NA	1.12 (0.80-1.56)	NA	0.91 (0.65-1.27)	NA	0.77 (0.54-1.11)	NA	0.81 (0.54-1.20)
Charlson Comorbidity Index score	NA	0.94 (0.90-0.99)	NA	1.33 (1.27-1.40)	NA	1.50 (1.42-1.59)	NA	1.21 (1.15-1.28)

Abbreviations: CVD, cardiovascular disease; ED, emergency department; NA, not applicable.

^a^ Model 1, unadjusted; model 2, adjusted for all variables listed.

^b^ Referent group is cancer.

Cause of death was strongly associated with hospice use and treatment intensity in our fully adjusted model. For example, patients who died of CVD were twice as likely as those with cancer to have multiple hospital visits (aOR, 1.78; 95% CI, 1.20-2.64) or ED visits (aOR, 2.00; 95% CI, 1.38-2.88) and 3 times as likely to undergo intensive procedures (aOR, 2.92; 95% CI, 1.86-4.60) but less likely to use hospice at the end of life (aOR, 0.25; 95% CI, 0.18-0.36) (Table 3). On the other hand, while dementia was associated with less hospice use (aOR, 0.62; 95% CI, 0.38-1.01) and more ED visits (aOR, 2.29; 95% CI, 1.39-3.79), there was no significant increase in the use of intensive procedures relative to those with cancer (aOR, 1.12; 95% CI, 0.48-2.59).

Interactions by race and cause of death were not significant for hospice use, multiple hospital visits, or intensive procedure use. A statistically significant interaction was detected for multiple ED visits, which suggests an increased association among Black patients with CVD or other illness compared with cancer (eTable in the Supplement). Relative to those dying of cancer, Black decedents with CVD or other illnesses had a 2-fold odds of multiple ED visits relative to White decedents.

## Discussion

Consistent with Brown et al,^28^ we documented extensive racial differences in end-of-life treatment, with Black decedents receiving less hospice care and having more ED visits, hospitalizations, and intensive treatments in the 6 months immediately preceding death compared with White decedents. Although these findings remained even when we accounted for cause of death and other clinical and demographic characteristics in the analysis, we found that racial differences in treatment intensity were especially pronounced among those with noncancer diagnoses.

Although this study was not able to assess whether treatment-intensity measures were aligned with patient and family care treatment preferences, we know that most individuals report that they want less invasive care at the end of life.^2,29^ Several possible explanations may underlie the higher intensity of care received by Black decedents in our study. First, other studies have documented that lack of trust in the medical system by Black patients is associated with a reduced willingness to forgo life-sustaining measures^30,31^ and an increased use of the ED for usual care.^32^ Second, poor communications between Black patients and health care professionals are documented and may be associated with differences in the intensity of health care service use.^33,34^ In addition, cultural and spiritual differences may play an important in role in choosing to forgo life-sustaining procedures.^35^ Black decedents may also have less access to higher-quality end-of-life care, including engagement in advance care planning in part owing to lower health literacy.^36^

Our study afforded us the opportunity to examine racial differences in the context of rigorously ascertained causes of death. Consistent with other findings,^37^ underlying cause of death was strongly associated with hospice use after controlling for other factors. Persons without cancer were far less likely to use hospice care relative to individuals with a cancer-related cause of death. Several possible explanations exist for the observed discrepancy in hospice use based on underlying cause of death. Health care professionals who provide care for many common noncancer illnesses may lack awareness about the utility of hospice services. Low use of hospice care by patients without cancer may also be associated with poor prognostic accuracy for many common noncancer illnesses, such as CVD or dementia. Cancer trajectories are more predictable than noncancer disease trajectories.^38^ Thus, it is easier for physicians to prognosticate when the life expectancy of a patient with cancer is 6 months or less, a criterion for hospice enrollment under the Medicare Hospice Benefit. Among cancer deaths, in fact, we did not find any racial differences in any marker of treatment intensity we examined, which suggests that the use of hospice care and more supportive care services may be more widely disseminated among those with advanced cancer, possibly reflecting the increased emphasis on end-of-life care for patients with cancer in the US health care system in recent years. Among decedents with dementia, we did not find any differences in hospice use, but we still saw a significant increase in the use of life-sustaining procedures among Black patients, a particularly troublesome finding given the lack of utility of these procedures at the end of life for this population.^39,40^

Despite an increased use of hospice care in the United States, there is a continued use of high-intensity treatments at the end of life,^41^ and significant differences by race and cause of death remain.^3^ Efforts to reduce disparities in the quality of end-of-life care must be prioritized. In particular, targeted efforts to increase advance care planning among Black and other racial minority populations should be expanded.^42^ The recent Centers for Medicare & Medicaid Services regulation to reimburse physicians and others for advance care planning discussions is another important step toward that goal.^43^ Reducing the stigma of hospice use through education and community outreach is critical.^44,45^ In particular, reducing disparities in hospice use in populations with noncancer causes of death will require improved prognostication, better patient-clinician communication, and rethinking current hospice outreach and enrollment practices.^46,47^

Using this national study with oversampling of Black participants, we had a large population to examine racial differences in end-of-life care. Furthermore, we had expert adjudication of the underlying cause of death and, hence, could examine differences in hospice use and other treatments by cause of death. We also included a marker of income. Disproportionate socioeconomic disadvantage is associated with some, but not all, of the differences in health care intensity at the end of life by race.^28^ Although our study was able to detect differences by race, larger studies are required to better examine interactions by race, educational level, and cause of death to understand the mechanisms by which disparities may exist.

### Limitations

Our findings should be interpreted with a number of potential limitations in mind. We were limited to decedents with Medicare (in particular, those who had fee-for-service Medicare 6 months before death). Those excluded were younger, more likely to be Black, and had lower income. Our results therefore may not be generalizable to all decedents. Although we were able to identify deaths due to cancer, CVD, and dementia, our dementia decedent subgroup was too small to capture meaningful differences. This group in particular requires further study given the growing prevalence of persons living with dementia and the need for better treatment at the end of life, including appropriate hospice care use. Dementia may also have been underdiagnosed in our sample of patients who did not have routine cognitive testing,^48^ with differential diagnosis by race. In addition, we grouped together multiple causes of death as “other illness.” Although we examined differences between Black and White decedents, we were unable to examine other racial groups that were excluded by design in REGARDS. Our work regarding differences in care may still be applicable to other disadvantaged populations. Furthermore, while we included careful adjustment of variables in models, there may still be residual confounding. Finally, we did not have detailed information on patient and family treatment preferences, whether treatment was concordant with care received, prognostic uncertainty, or overall satisfaction with the care received. These factors are likely to vary by disease status and should be explored further.

## Conclusions

Our work suggests that Black decedents undergo more intensive treatments at the end of life and are less likely to use hospice services relative to White decedents. More sustained efforts must be made to reduce disparities in end-of-life care through efforts to better educate and train health care professionals and to promote the discussion of personal values and treatment preferences for the end of life in Black populations.

## Notes

Supplement. **eFigure.** Derivation of Analytic Sample **eTable.** Interaction of Race and Cause of Death on End-of-Life Treatment Intensity
