# Supplementary material for: Evaluation of Racial Disparities in Hospice Use and End-of-Life Treatment Intensity in the REGARDS Cohort
Source: JAMA Netw Open. 2020 Aug 24;3(8):e2014639. doi: 10.1001/jamanetworkopen.2020.14639 (PMC7445597; doi:10.1001/jamanetworkopen.2020.14639)
Supplement: Supplement. — eFigure. Derivation of Analytic Sample eTable. Interaction of Race and Cause of Death on End-of-Life Treatment Intensity [file jamanetwopen-3-e2014639-s001.pdf]

Supplementary Online Content

Ornstein KA, Roth DL, Huang J, et al. Evaluation of racial disparities in hospice use and end-of-life treatment intensity in the REGARDS cohort. *JAMA Netw Open.* 2020;3(8):e2014639. doi:10.1001/jamanetworkopen.2020.14639

eFigure. Derivation of Analytic Sample
eTable. Interaction of Race and Cause of Death on End-of-Life Treatment Intensity

This supplementary material has been provided by the authors to give readers additional information about their work.

© 2020 Ornstein KA et al. *JAMA Network Open.*

eFigure. Derivation of Analytic Sample

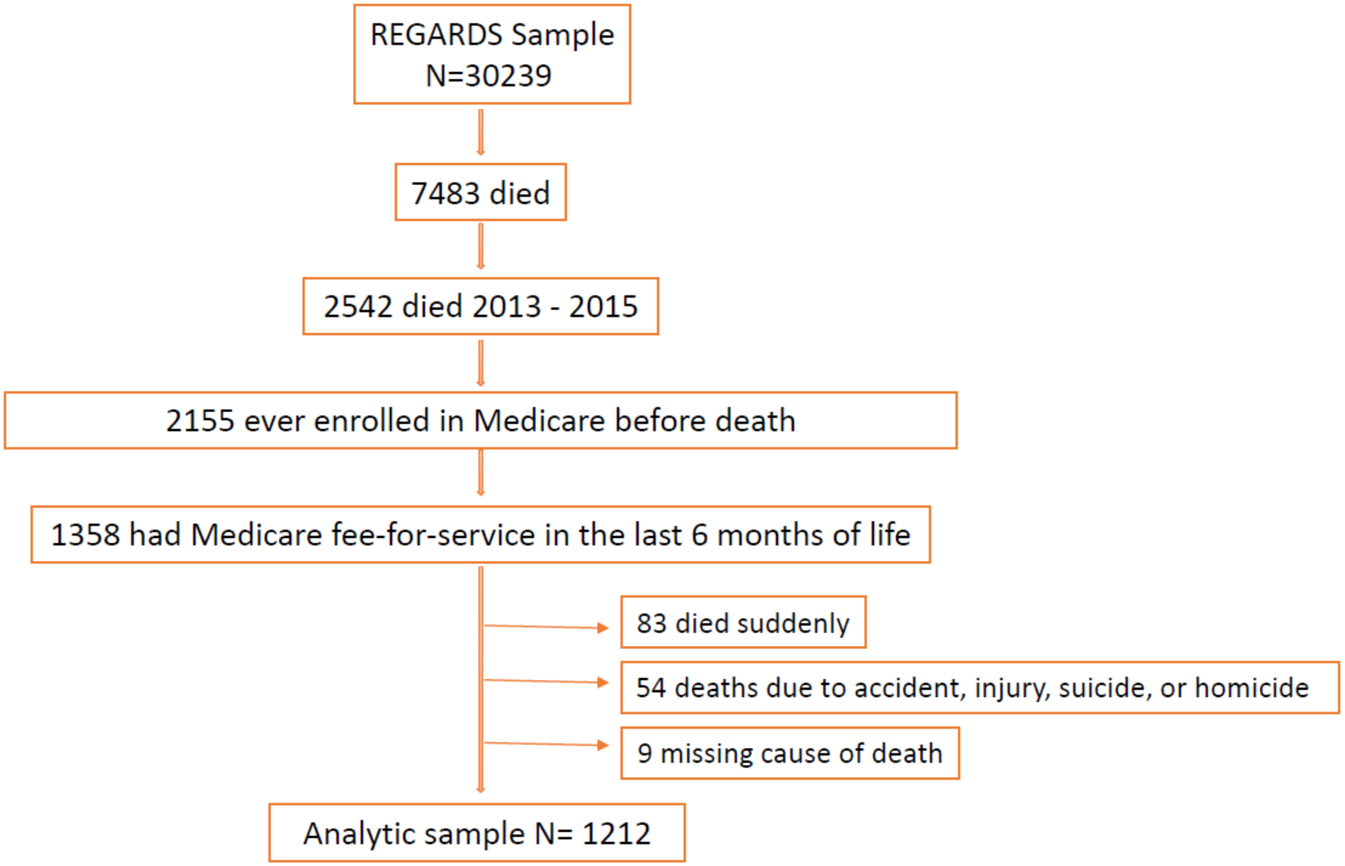

© 2020 Ornstein KA et al. *JAMA Network Open.*

eTable. Interaction of Race and Cause of Death on End-of-Life Treatment Intensity

Variable	Hospice use ≥ 3 days		≥2 ED visits		≥2 hospital visits		Any Intensive Procedures	
	**Estimate (standard error)**	***p*-value**	**Estimate (standard error)**	***p*-value**	**Estimate (standard error)**	***p*-value**	**Estimate (standard error)**	***p*-value**
Race, Black vs. White	-0.35 (0.26)	0.18	-0.21 (0.26)	0.43	0.35 (0.26)	0.18	0.42 (0.33)	0.21
COD								
CVD vs. Cancer	-1.11 (0.20)	<0.001	-0.20 (0.20)	0.31	-0.41 (0.21)	0.04	0.19 (0.26)	0.48
Dementia vs. Cancer	-0.16 (0.27)	0.55	-0.29 (0.26)	0.27	-0.91 (0.31)	<0.01	-1.90 (0.74)	0.01
Other illness vs. Cancer	-1.15 (0.18)	<0.001	-0.13 (0.17)	0.44	-0.09 (0.18)	0.63	0.56 (0.23)	0.01
Race * COD								
Black and CVD	-0.03 (0.37)	0.93	1.11 (0.36)	<0.01	0.52 (0.36)	0.15	0.62 (0.43)	0.15
Black and dementia	0.32 (0.48)	0.51	0.57 (0.48)	0.23	0.20 (0.52)	0.70	1.58 (0.91)	0.08
Black and other illness	-0.29 (0.34)	0.39	0.93 (0.33)	<0.01	0.26 (0.33)	0.42	0.54 (0.39)	0.17

© 2020 Ornstein KA et al. *JAMA Network Open.*
